# Identifying a whole‐brain connectome‐based model in drug‐naïve Parkinson's disease for predicting motor impairment

**DOI:** 10.1002/hbm.25768

**Published:** 2021-12-31

**Authors:** Haoting Wu, Cheng Zhou, Xueqin Bai, Xiaocao Liu, Jingwen Chen, Jiaqi Wen, Tao Guo, Jingjing Wu, Xiaojun Guan, Ting Gao, Luyan Gu, Peiyu Huang, Xiaojun Xu, Baorong Zhang, Minming Zhang

**Affiliations:** ^1^ Department of Radiology The Second Affiliated Hospital, Zhejiang University School of Medicine Hangzhou China; ^2^ Department of Neurology The Second Affiliated Hospital, Zhejiang University School of Medicine Hangzhou China

**Keywords:** brain connectome, motor impairment, Parkinson's disease, predictions, resting‐state fMRI

## Abstract

Identifying a whole‐brain connectome‐based predictive model in drug‐naïve patients with Parkinson's disease and verifying its predictions on drug‐managed patients would be useful in determining the intrinsic functional underpinnings of motor impairment and establishing general brain–behavior associations. In this study, we constructed a predictive model from the resting‐state functional data of 47 drug‐naïve patients by using a connectome‐based approach. This model was subsequently validated in 115 drug‐managed patients. The severity of motor impairment was assessed by calculating Unified Parkinson's Disease Rating Scale Part III scores. The predictive performance of model was evaluated using the correlation coefficient (*r*_true_) between predicted and observed scores. As a result, a connectome‐based model for predicting individual motor impairment in drug‐naïve patients was identified with significant performance (*r*_true_ = .845, *p* < .001, *p*_permu_ = .002). Two patterns of connection were identified according to correlations between connection strength and the severity of motor impairment. The negative motor‐impairment‐related network contained more within‐network connections in the motor, visual‐related, and default mode networks, whereas the positive motor‐impairment‐related network was constructed mostly with between‐network connections coupling the motor‐visual, motor‐limbic, and motor‐basal ganglia networks. Finally, this predictive model constructed around drug‐naïve patients was confirmed with significant predictive efficacy on drug‐managed patients (*r* = .209, *p* = .025), suggesting a generalizability in Parkinson's disease patients under long‐term drug influence. In conclusion, this study identified a whole‐brain connectome‐based model that could predict the severity of motor impairment in Parkinson's patients and furthers our understanding of the functional underpinnings of the disease.

## Graphical abstract

The whole‐brain connectome‐based model was able to predict the severity of motor impairment among drug‐naive patients and further confirmed among independent drug‐managed group, suggesting generalizability in Parkinson's disease (PD) patients with lasting drug influence. Within‐network dysfunction of motor, visual‐related, and default mode networks play an important role in PD motor impairment. Higher connections coupling motor and non‐motor networks might demonstrate a compensatory mechanism to overcome motor dysfunction.

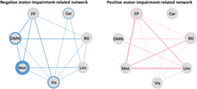

**Abbreviations**
CPM: connectome‐based predictive model
FD: frame‐wise displacement
H–Y stage: Hoehn and Yahr stage
LEDD: levodopa equivalent daily dose
LOOCV: leave‐one‐out cross‐validation
MMSE: Mini‐Mental State Examination
MSE: mean squared error
PD: Parkinson's disease
rs‐fMRI: resting‐state functional magnetic resonance imaging
UPDRS III: Unified Parkinson's Disease Rating Scale Part III

## 1 INTRODUCTION

Parkinson's disease (PD) is the second most common neurodegenerative disease in aging populations (Hayes, 2019). In its most classical manifestation, PD is characterized by progressive motor impairment (Kalia & Lang, 2015), which results from abnormalities of the basal ganglia circuits due to the death of dopaminergic neurons in the pars compacta of the substantia nigra (Kalia, Brotchie, & Fox, 2013). However, pathophysiological changes outside of the basal ganglia are widely acknowledged to also have significant roles in modulating motor loops (Bartels & Leenders, 2009; Guan et al., 2019; Kalia et al., 2013). Moreover, complex within‐network segregation and between‐network coupling might significantly contribute to motor disorder, but in such a large‐scale brain network view, the potential network underpinning for PD is not well investigated, and little is known about the intricate interactions between or within distinct networks.

Resting‐state functional magnetic resonance imaging (rs‐fMRI) has provided an approach to studying the central processing of motor impairment in vivo. In numerous studies, rs‐fMRI has been used to identify associations between motor impairment and functional connections in classical motor regions such as the basal ganglia and motor cortex (Hacker, Perlmutter, Criswell, Ances, & Snyder, 2012; Nachev, Kennard, & Husain, 2008), as well as in non‐motor regions including the frontoparietal and visual networks and the limbic system (Gilat et al., 2018; Kann, Chang, Manza, & Leung, 2020; Tessitore et al., 2012; Vervoort et al., 2016). However, the findings vary greatly. One of the main concerns is that PD patients recruited into these studies have already been exposed to dopaminergic medication, which would lead to heterogeneous reorganizations of brain function in order to preserve motor behavior (Krismer & Seppi, 2021; Tahmasian et al., 2015). This important influence has been widely ignored. Therefore, we hypothesized that studies in drug‐naïve patients are a priority for investigating the intrinsic and complex interactions between/within district networks that relate to motor impairment, and might provide a robust prediction of motor impairment when the influence of medication is subsequently taken into consideration.

Moreover, because the neurodegenerative process acting on the human brain has little consensus between individuals, progresses along different trajectories, and is complicated by various pathophysiological factors, more attention has been paid to personal brain organization. Finding a link between an individual functional connectome and behavioral measurements can maximally reduce the bias inherent in population variation, and the resulting brain–behavior associations observed would be more robust and generalized (Tessitore, Cirillo, & De Micco, 2019). Thus, a connectome‐based predictive modeling (CPM) approach has been newly introduced to predict behavior at the individual level by using large‐scale network functional connectivity in a machine‐learning framework. This has been used to investigate the complex mechanisms underlying mental and cognitive disorders (Gao et al., 2020; Ren et al., 2021; Yu et al., 2020), as well as in predicting outcomes after deep brain stimulation in PD patients (Shang, He, Ma, Ma, & Li, 2020). Therefore, by taking advantage of a CPM framework built on informative large‐scale network connections, a novel predictive model would be constructed for identifying intrinsic network patterns for drug‐naïve patients, which might have robust performance in predicting motor impairment.

Hence, this study aimed to construct a whole‐brain connectome model that can predict motor impairment in PD patients. In order to reveal the disease‐intrinsic functional underpinnings free of the effects of dopaminergic medication, we constructed a predictive model on drug‐naïve patients and tested its performance in an independent group of drug‐managed PD patients to check its reliability.

## 2 METHODS

### 2.1 Participant enrollment and evaluation

All patients signed informed consent forms in accordance with the approval of the Medical Ethics Committee of the Second Affiliated Hospital of Zhejiang University School of Medicine.

Two hundred PD patients were initially recruited to this study. The diagnosis of PD was made by a senior neurologist (B. R. Z.) according to the United Kingdom Parkinson's Disease Society Brain Bank criteria (Hughes, Daniel, Kilford, & Lees, 1992). Twenty‐four patients were excluded on the basis of having either (a) cerebrovascular disorders, including previous stroke, history of head injury, or other neurological diseases (*N* = 14); or (b) cognitive impairment based on the Mini‐Mental State Examination (MMSE), estimated by the criteria applicable to the Chinese population (MMSE score ≤ 17 for illiterate patients, ≤20 for grade‐school literates, and ≤23 for junior high school and higher education literates [*N* = 10]) (Katzman et al., 1988; M. Y. Zhang et al., 1990). A final total of 176 PD patients were enrolled in this study, comprising 49 drug‐naïve and 127 drug‐managed patients. Drug‐managed PD patients underwent clinical assessment on the morning after all dopamine replacement therapy was withdrawn overnight (at least 12 hr into their “drug‐off status”). Basic demographic information, including age, gender, level of education, and duration of disease, and neurological and psychiatric scales including Unified Parkinson's Disease Rating Scale Part III (UPDRS III) score, Hoehn and Yahr stage (H–Y stage), and MMSE score were obtained for all patients. The total levodopa equivalent daily dose (LEDD) (Tomlinson et al., 2010) and duration of treatment were recorded for drug‐managed patients.

### 2.2 Image acquisition and preprocessing

#### 2.2.1 Image acquisition

All imaging data were acquired on a 3.0 T magnetic resonance imaging (MRI) scanner (Discovery MR750, GE Healthcare). MRI scanning of each drug‐managed patient was carried out in the drug‐off status. The head of each participant was stabilized with foam pads, and earplugs were provided to reduce audible noise during scanning.

rs‐fMRI data were acquired using gradient recalled echo–echo planar imaging sequence: echo time = 30 ms; repetition time = 2,000 ms; flip angle = 77°; field of view = 240 × 240 mm^2^; matrix = 64 × 64; slice thickness = 4 mm; slice gap = 0 mm; number of slices = 38 (axial); time points = 205. Structural T1‐weighted images were acquired using a fast‐spoiled gradient‐recalled sequence: echo time = 3.036 ms; repetition time = 7.336 ms; inversion time = 450 ms; flip angle = 11°; field of view = 260 × 260 mm^2^; matrix = 256 × 256; slice thickness = 1.2 mm; number of slices = 196 (sagittal). All the sequence fields of view covered the whole brain, including the cerebrum, cerebellum, and brain stem.

#### 2.2.2 Image preprocessing

Rs‐fMRI data processing was carried out using Statistical Parametric Mapping (SPM 12, https://www.fil.ion.ucl.ac.uk/spm/) and Data Processing Assistant for Resting State fMRI (DPABI_V3.1_180801, http://www.rfmri.org/) (Yan, Wang, Zuo, & Zang, 2016). In an initial step, the first 10 volumes of the functional time series were deleted to utilize the MRI signal at equilibrium. The remaining images underwent slice timing for interval scanning, realignment, and normalization to the standard MNI space through T1 image segmentation. Next, spatial smoothing with a Gaussian kernel of 6 × 6 × 6 mm full width at half‐maximum, detrending, covariate regression (Friston 24‐motion parameters, mean signals of white matter and cerebrospinal fluid), and band‐pass temporal filtering (0.01–0.1 Hz) were sequentially applied to the remaining volumes.

#### 2.2.3 Control of head motion

To account for the effect of head motion on the rs‐fMRI analysis, volumes with mean frame‐wise displacement (FD) ≥ 0.2 mm were removed, and the remaining volumes were used for network construction. Then, 14 individuals—2 drug‐naïve and 12 drug‐managed patients—having <4 min (120 volumes) of data after scrubbing were excluded from the following analysis (Jenkinson, Bannister, Brady, & Smith, 2002; Parkes, Fulcher, Yücel, & Fornito, 2018). Consequently, a total of 162 PD patients were enrolled in this study, comprising 47 drug‐naïve and 115 drug‐managed patients. To verify that neither the observed nor the predicted scores were correlated with head‐motion, correlation coefficients were calculated between the mean FD and observed and predicted scores, respectively. To further control for possible head‐motion effects, we also applied a prediction analysis with the mean FD as an additional nuisance variable within the candidate connection selection process described in Section 2.3.1.

#### 2.2.4 Functional network construction

Consistent with previous CPM‐based studies, network nodes were defined using the 268‐region‐of‐interest functional brain atlas (Shen, Tokoglu, Papademetris, & Constable, 2013). This atlas covers the whole brain, including cortical, subcortical, and brainstem structures. The whole‐brain functional connection matrix was constructed for each patient in the MNI space. The mean time series of each node was extracted by averaging the time series of all voxels in each defined node. The functional connection was then calculated as the Pearson correlation coefficient (*r*) between the mean time series of each pair of nodes. Both positive and negative correlation coefficients were included to construct the connection matrix. A Fisher's *r*‐to‐*z* transformation was then used to normalize the correlation coefficients, and the resulting 268 × 268 matrix for each participant was utilized for the subsequent CPM analysis. Each element of the matrix represented the strength of connection between two nodes.

### 2.3 Connectome‐based model construction and evaluation in drug‐naïve patients

**FIGURE 1:**
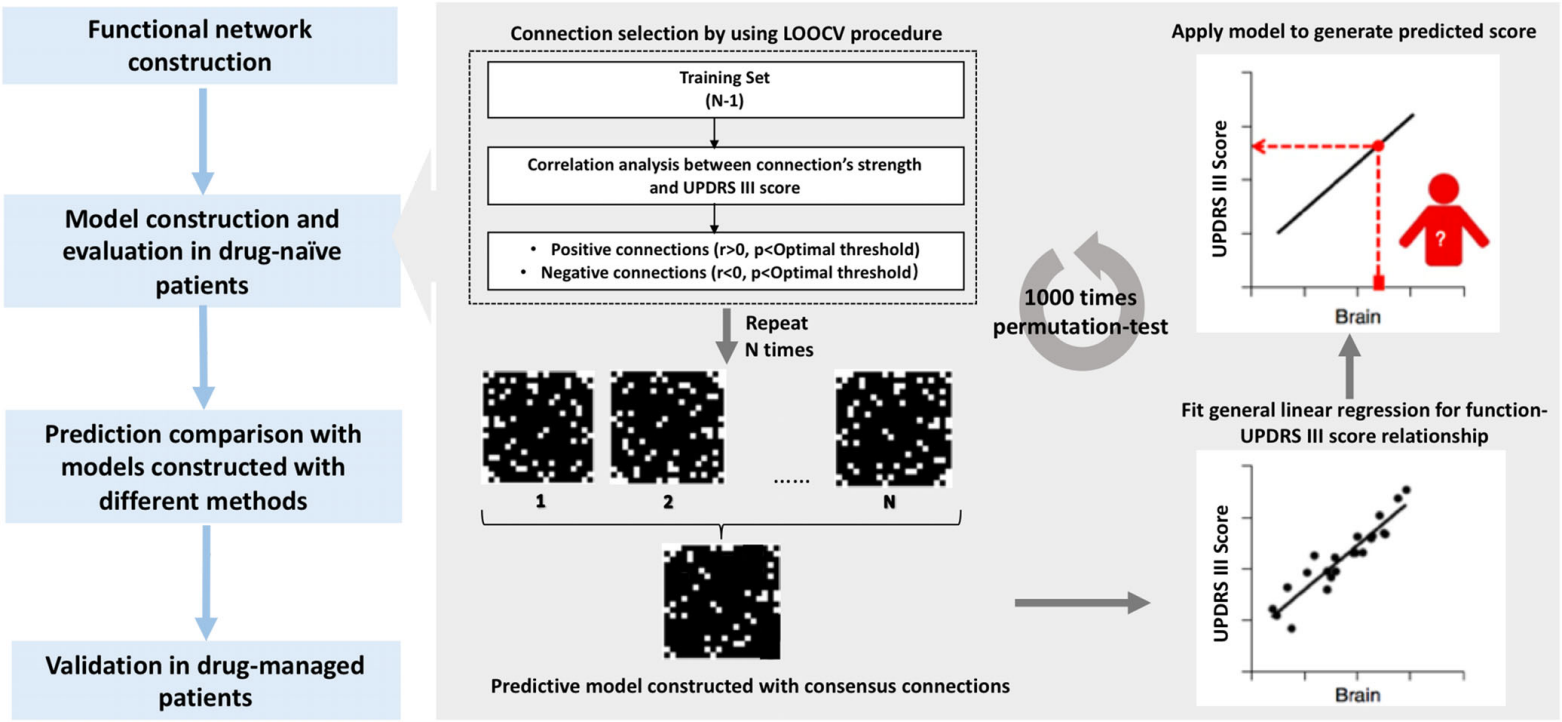
Workflow for identifying a whole‐brain connectome‐based model for predicting motor impairment in PD. Model M was first constructed and evaluated among drug‐naïve PD patients. Its predictive performance was further validated among drug‐managed PD patients for reliability checking. LOOCV, leave‐one‐out cross‐validation; PD, Parkinson's disease; UPDRS III, the Unified Parkinson's Disease Rating Scale Part III

A flowchart for the construction of the connectome‐based model and its evaluation is shown in Figure 1. All processes were performed by applying free scripts in MATLAB (R2020b for Windows, MathWorks). These scripts are available at https://www.nitrc.org/projects/bioimagesuite/.

#### 2.3.1 Selection of candidate connections by using a leave‐one‐out cross‐validation procedure

Acknowledging the relatively small number of drug‐naïve patients (*N* = 47), a leave‐one‐out cross‐validation (LOOCV) procedure was used to select candidate connections (Rosenberg et al., 2016; Scheinost et al., 2019). The LOOCV procedure was repeated iteratively. In each iteration, one patient was removed from the training set and data for the remaining *N* − 1 patients were used for testing according to the following steps. First, the correlation between the strength of each connection and the observed UPDRS III score was assessed. In this step, Spearman's analysis was applied since the observed scores in this study were not normally distributed (Kolmogorov–Smirnov test, *p* < .05) (Shen et al., 2017). A partial correlation analysis was also conducted to ensure that the constructed model captured meaningful connection alternation associated with motor impairment (Scheinost et al., 2019). Three nuisance variables correlating with the phenotypic measure or neuroimaging data were included: age (significantly correlated with UPDRS III scores), duration (significantly correlated with UPDRS III scores), and gender (shown by Zhang, Dougherty, Baum, White, and Michael (2018) to affect functional connections). Next, the connections were selected based on the significance of the correlation between the connection strength and UPDRS III score. The significance threshold of *p* value was optimized to afford the best predictive performance (detailed in Section 2.3.4). Finally, all selected connections were categorized as either positive connections (connections for which strength indexed with higher UPDRS III score and severe motor impairment) or negative connections (the strength of which indexed with lower UPDRS III score and milder motor impairment) according to their correlation coefficients with observed scores. The above‐mentioned steps were repeated *N* times (*N* = 47) until all patients had been excluded.

#### 2.3.2 Model construction with consensus connections and prediction evaluation

After the LOOCV procedure was performed, 47 sets of candidate connections were obtained. Owing to the nature of cross‐validation, a slightly different set of candidate connections can be selected in different iterations. To reduce potential variation, the connections finally utilized for model construction should be selected in each iteration, and are termed “consensus connections.” These connections had the highest reliability among all candidate connections. All positive consensus connections and negative consensus connections were marked to construct respective binary masks. These two masks were then applied to each patient's own matrix to calculate the sum strengths of the positive and negative consensus connections. Summed strengths of positive and negative consensus connections were then fit with general linear regression to build a relationship with the observed score. The predicted score of each patient could be calculated by applying the constructed linear model with the following formula:

$$\text{predicted score}=a_{1}\times x_{1}+a_{2}\times x_{2}+b$$

where $x_{1}$ is the sum of the strengths of positive consensus connections and $x_{2}$ is the sum of the strengths of negative consensus connections.

The performance of the constructed model was evaluated by calculating the Spearman correlation coefficient (*r*_true_) and the mean squared error (MSE) between observed and predicted UPDRS III scores. The values of a correlation coefficient and the MSE are usually dependent, that is, a higher correlation implies lower MSE and vice versa. A lower MSE value means that the difference between the predicted and observed scores is smaller (Shen et al., 2017). The significance of the constructed model was further tested by applying the 1,000‐permutation test (Ren et al., 2021), which involved randomly shuffling the UPDRS III score and repeating the above processes 1,000 times. The significance of the permutation test was analyzed by calculating the percentage of sampled permutations that were greater or equal to the *r*_true_ value (*p*_permu_); *p*_permu_ < .05 was considered statistically significant.

#### 2.3.3 Prediction comparison among connectome‐based models constructed using different methods

We sought to determine (a) whether a model constructed with a combination of positive and negative connections performs better than a model constructed with only positive or negative connections, and (b) whether models constructed with consensus connections selected in all iterations (representing highest reliability) perform better than models constructed with candidate connections in each iteration (representing less reliability). To these ends, the predictive power of model M was compared with other three models: model M1 constructed with positive consensus connections, model M2 constructed with negative consensus connections, and model M3 in which predicted scores were generated from each LOOCV iteration. Optimization of the threshold and permutation testing were also applied during the construction of M1, M2, and M3. Thus, the thresholds of these four models can vary. The processes for constructing models M1, M2, and M3 are detailed in the supporting information. To determine whether the predictive power of these models was significantly different from model M, Steiger's *Z* test was used to compare the *r*_true_ value of model M with those of the other three (Rosenberg et al., 2016).

#### 2.3.4 Optimal threshold value

For selection of candidate connections, rather than applying an arbitrary *p* value threshold as did a previous study (Rosenberg et al., 2016), predictive ability was compared using different *p* value cutoffs. These thresholds were evaluated by repeating the above process 50 times using *p* values ranging from .05 to .001, with intervals of .001. The *p* value that afforded the highest *r*_true_ value (the correlation coefficient between predicted and observed UPDRS III scores) was selected and used in construction of the model.

### 2.4 Model validation in drug‐managed patients

Finally, the model with the best predictive ability among the above‐mentioned four was further validated in drug‐managed patients. Correlation coefficient *r* and the MSE between observed and predicted scores were also calculated. The significance of *r* was calculated using standard parametric conversion, and *p* < .05 was considered statistically significant.

### 2.5 Functional network anatomy of the constructed connectome model

#### 2.5.1 Grouping nodes into seven functional networks

The 268 nodes were divided into seven canonical functional networks according to anatomical order and previous studies (Lake et al., 2019; Shen et al., 2013), which were: frontoparietal (63 nodes), default mode (20 nodes), motor (50 nodes), visual‐related (45 nodes), limbic (30 nodes), basal ganglia (29 nodes), and cerebellum (31 nodes) networks. The visual‐related network comprised visual I, visual II, and visual association networks. A map of these seven networks is shown in the supporting information (Figure S1). Connections within each network (“within‐network connections”) were calculated by summarizing the number of connections between nodes of that same network. Connections between two distinct networks (“between‐network connections”) were calculated by summarizing the number of connections between nodes from different networks.

#### 2.5.2 Contribution of each functional network

We weighted each functional network's contribution by calculating the sum of positive consensus and negative consensus connections belonging to it; a greater number of connections indicate a greater contribution. To further assess the importance of each functional network to the prediction of motor impairment, we computationally “lesioned” the model to exclude connections from it. Accordingly, in an iterative analysis, we masked the connection matrix to exclude connections that appeared in one of the seven functional networks. For example, after excluding connections in the frontoparietal networks, which contained 63 nodes, a 205 × 205 matrix rather than a 268 × 268 matrix was submitted for analysis. Models with lesioned matrices, termed “lesioned models,” were first constructed and evaluated among drug‐naïve patients and then validated with drug‐managed patients. The predictive ability of each lesioned model was evaluated according to the correlation between the predicted and observed UPDRS III scores, and the significance of the prediction was confirmed by permutation test in drug‐naïve patients. The predictive ability of the lesioned model was compared with the original model by using Steiger's test. All *p* values were adjusted using a 14‐comparison Bonferroni correction.

#### 2.5.3 Two connection patterns from the connectome model

To better understand the relationships between motor impairment in PD and various within‐ and between‐functional connections, we divided the connectome model into two connection patterns. One pattern that contained all the negative consensus connections was called the “negative motor‐impairment‐related network.” The other pattern, containing all the positive consensus connections, was called the “positive motor‐impairment‐related network.” The characteristics of within‐ and between‐network connections were analyzed by summing the consensus connections using the above‐mentioned seven canonical functional networks in the positive and negative motor‐impairment‐related networks. To control the effect of variation in network size, the proportion of the connections within and between networks was also calculated by dividing the actual number of connections by the total number of possible connections (Gao et al., 2020).

### 2.6 Statistical analyses

The clinical characteristics of drug‐naïve and drug‐managed patients were analyzed by using SPSS software (version 25). A Kolmogorov–Smirnov test was applied to identify the normal distribution of continuous variables. Differences in normally distributed variables between the two groups were evaluated with the two‐sample *t* test; otherwise, the Mann–Whitney test was conducted. Differences for qualitative variables were compared using the chi‐square test. Statistical analyses of model construction, validation, and evaluation were performed in MATLAB (R2020b, MathWorks); details are given in the respective sections above. Unless stated otherwise, a two‐sided *p* value <.05 was considered significant.

## 3 RESULTS

### 3.1 Characteristics of enrolled patients

A total of 162 PD patients were enrolled in this study, including 47 drug‐naïve and 115 drug‐managed patients. The characteristics of the patients in these two groups are summarized in Table 1. With the exception of treatment status (LEDD and duration of treatment), drug‐managed patients had a significantly longer duration of disease (*p* = .005), higher H–Y stage (*p* < .001), and higher UPDRS III score (*p* = .002) compared with drug‐naïve patients. No significant differences in age, gender, MMSE score, and level of education were observed between the two groups. Spearman correlation analysis demonstrated that UPDRS III scores of drug‐naïve patients were significantly associated with age (*r* = .404, *p* = .004) and disease duration (*r* = .527, *p* < .001), which had been controlled by partial analysis in the process of candidate connection selection.

**TABLE 1:** Patients' characteristics

Patient characteristic	Drug‐naïve group (*N* = 47)	Drug‐managed group (*N* = 115)	*p*‐Value
Gender (male:female)	22:25	65:50	.061
Age (years, mean ± *SD*)	57.72 ± 10.085	60.44 ± 9.581	.109
Duration of disease (years, median (range))	1.82 (0.08–10.25)	3.47 (0.08–26.37)	.005*
UPDRS III score (median (range))	18 (4–68)	19 (3–66)	.002*
H–Y stage (median (range))	2 (1–3)	2.5 (1–5)	<.001*
LEED (median (range))	—	425 (25–1,300)	NA
Duration of treatment (years, median (range))	—	2.04 (0.04–26.37)	NA
MMSE (median (range))	28 (19–30)	28 (17–30)	.497
Education (median (range))	9 (0–18)	9 (0–18)	.459

*Note*: *p* < .05 was considered statistically significant (annotated with *).

Abbreviations: H–Y stage, Hoehn and Yahr stage; LEDD, levodopa equivalent daily dose; MMSE, Mini‐Mental State Examination; NA, not available; UPDRS III, the Unified Parkinson's Disease Rating Scale Part III.

### 3.2 Constructing and evaluating models in drug‐naïve patients

#### 3.2.1 Optimal threshold for selecting connections

The optimal threshold for maximizing the *r*_true_ values of models M, M1, M2, and M3 was identified after repeating the model construction process with connection‐selection thresholds of *p* values ranging from .05 to .001. In addition, the MSE value under each threshold within these four models was calculated to further evaluate the predictive accuracy. The optimal *p* value thresholds of M, M1, M2, and M3 were .009, .008, .001, and .001, respectively. In general, there appeared to be a consistent inverse relationship between *r*_true_ and the MSE, which meant that the *p* value associated with the highest *r*_true_ value was highly similar to that associated with a low MSE value. The *r*_true_ and MSE values across the tested range of *p* values are shown in Figure S2.

#### 3.2.2 Prediction evaluation and model comparison

**FIGURE 2:**
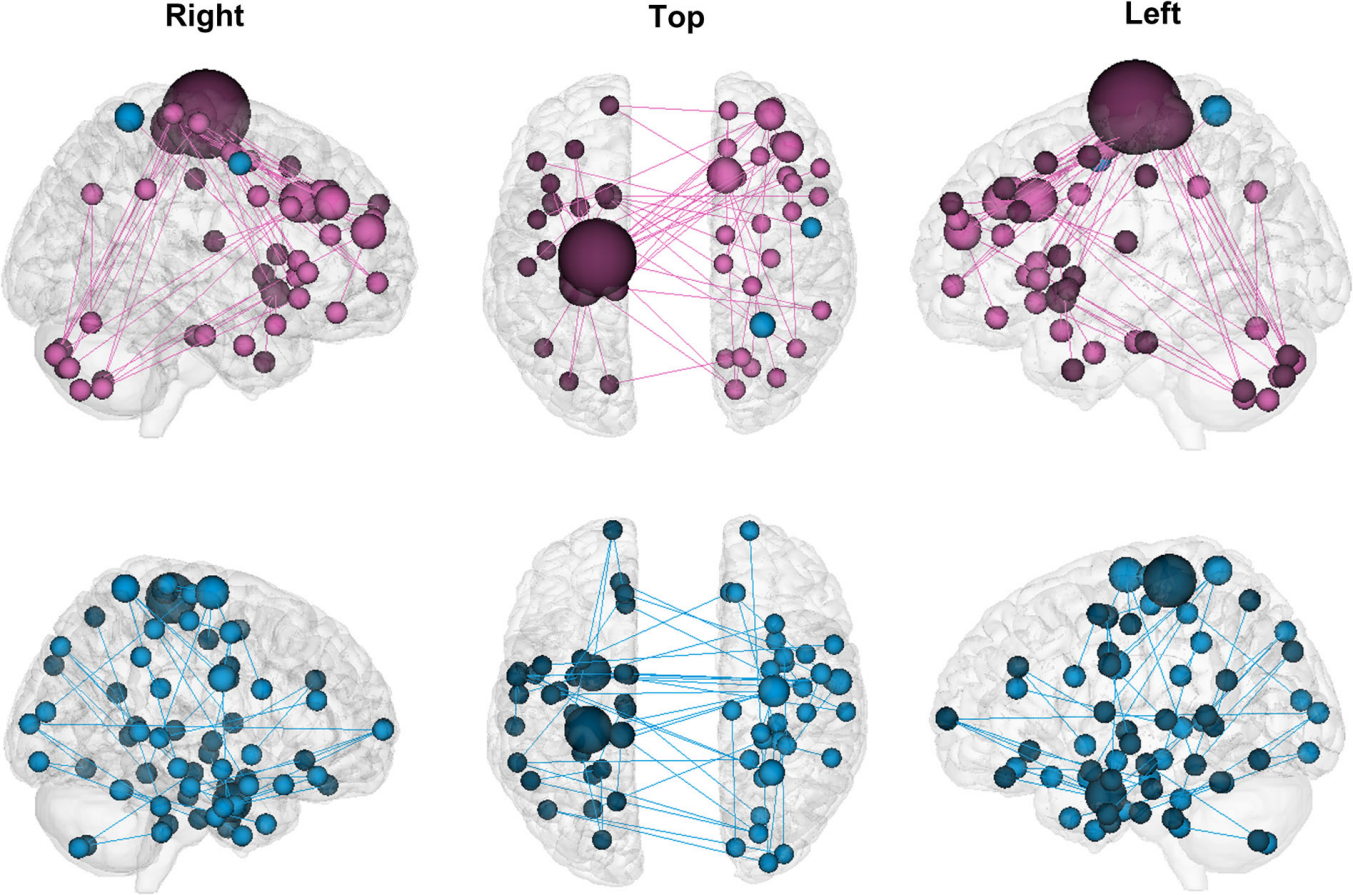
The constructed model M contained 57 positive consensus connections (pink) and 58 negative consensus connections (blue). Connectivity figures were created using the tool available at http://bisweb.yale.edu/connviewer/

**FIGURE 3:**
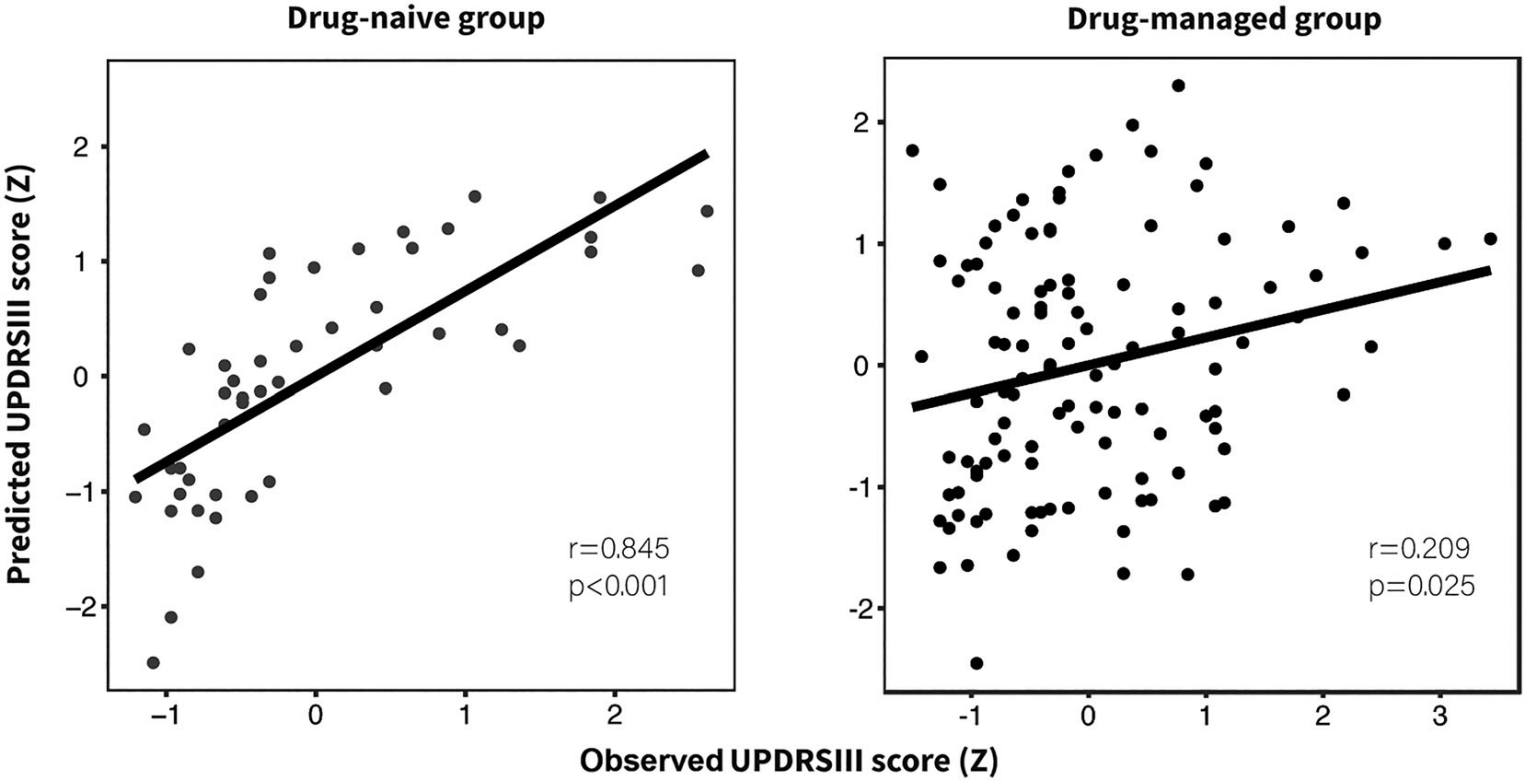
The constructed model M could predict individual motor impairment severity of drug‐naïve and drug‐managed patients. Both predicted and observed scores were standardized for visualization. UPDRS III, the Unified Parkinson's Disease Rating Scale Part III

There were 115 consensus connections selected to construct model M, including 58 negative consensus connections and 57 positive consensus connections (Figure 2). The strengths of positive and negative consensus connections significantly correlated with UPDRS III scores (positive connections: *r* = .712, *p* < .001; negative connections: *r* = −.661, *p* < .001; Figure S3). The highest sum of selected connections was obtained for model M, representing 0.32% of the sum (35,778) of whole‐brain connections. The significant correlation between the observed and predicted UPDRS III scores demonstrated that the constructed model could predict the severity of motor impairment in individual drug‐naïve PD patients (*r*_true_ = .845, *p* < .001, *p*_permu_ = .002, MSE = 137.57; Figure 3, drug‐naïve group). After controlling for clinical characteristics (including age, gender, disease duration, H–Y stage) within a partial correlation analysis, the predicted scores remained significantly associated with the observed scores (*r* = .763, *p* < .001).

Compared with models M1, M2, and M3, model M had a significantly higher *r*_true_ value (Table 2 and Figure S4). Furthermore, model M contained all consensus connections selected by M1 and M2. These results suggest that model M contained reliable and valuable information reflecting the core functional underpinnings associated with motor impairment, which afford the best predictive performance in the drug‐naïve group.

**TABLE 2:** Comparison of predictions from connectome‐based models

Model	*r*_true_ (*p*, *p*_permu_)	MSE	Steiger's *Z* value	*p*‐Value
M	.845 (<.001, .002)	137.57	—	—
M1	.712 (<.001, .014)	182.83	2.63	.008*
M2	.711 (<.001, .012)	159.95	2.39	.017*
M3	.528 (<.001, .002)	248.69	4.44	<.001*

*Note*: *r*_true_ is the true predictive correlation coefficient between observed and predict scores; *p*_permu_ is the *p* value obtained from permutation test (1,000 times); *p < .05 was considered statistically significant.

Abbreviation: MSE, mean squared error.

#### 3.2.3 Head motion control

The head motion of each patient, which was evaluated by calculating mean FD, was not significantly associated with either observed scores (*r* = .031, *p* = .837) or the predicted scores generated with model M (*r* = .045, *p* = .762). Furthermore, in combining the mean FD as an additional nuisance variable when selecting candidate connections, the constructed model remained predictive for drug‐naïve patients (*r*_true_ = .835, *p* < .001, *p*_permu_ = .001, MSE = 135.73). The predictive performance of this model was not significantly different from the original (Steiger's *Z* value = 1.936, *p* = .053). In addition, the common connections were highly overlapped with the network of model M after controlling for head motion (percentage overlap: higher‐UPDRS III score network 94.7%, lower‐UPDRS III score 91.4%). These results suggest that head motion did not have significant confounding effects on our principal results.

### 3.3 Validating the constructed model in drug‐managed patients

Model M significantly predicted UPDRS III score in the independent drug‐managed group (*r* = .209, *p* = .025, MSE = 182.96; Figure 3). This result remained stable after introducing head motion as a nuisance variable in the candidate connection selection process (*r* = .219, *p* = .019, MSE = 182.17) and did not significantly differ from the original (Steiger's *Z* value = 0.626, *p* = .535). These results demonstrated that head motion did not significantly affect the predictions of model M in drug‐managed patients. Furthermore, after controlling clinical characteristics (including age, gender, disease duration, H–Y stage, LEDD, and duration of treatment) within partial correlation analysis, the predicted scores generated with model M remained significantly associated with the observed scores (*r* = .214, *p* = .025).

### 3.4 A hybrid model combining clinical factors with the constructed model M

The value of using the clinical factors age, gender, disease duration, and H–Y stage for prediction were evaluated by adding them into model M. By combining the sum of positive consensus connections and the sum of negative consensus connections with these four clinical factors, the hybrid model successfully predicted UPDRS III score in both drug‐naïve (*r*_true_ = .961, *p* < .001, *p*_permu_ < .001) and drug‐managed groups (*r*_true_ = .459, *p* < .001). The significant improvement of predictions detected in both groups demonstrated the value added by including clinical factors (drug‐naïve group: 0.845 vs. 0.961, *p* < .001, drug‐managed: 0.209 vs. 0.459, *p* < .001).

### 3.5 Analysis of functional network anatomy

#### 3.5.1 Contribution of each functional network to prediction of motor impairment

By summing positive and negative consensus connections together, we found that the motor network contributed predominantly, followed by the frontoparietal and limbic networks. After controlling for network size, the results consistently showed that the motor, limbic, the frontoparietal networks were the top three contributors to the connectome model (Figure S5). Next, we tested the importance of each individual functional network for predicting motor impairment by constructing lesioned models (Table 3). Compared with the whole‐brain connectome model, in the drug‐naïve group, the predictive power of the lesioned model was reduced after excluding visual‐related (Steiger's *Z* value = 2.246, *p* = .025) and frontoparietal (Steiger's *Z* value = 2.149, *p* = .032) networks. In the drug‐managed group, the predictive power was reduced after excluding the basal ganglia network (Steiger's *Z* value = 2.232, *p* = .026). The results of the Steiger's test did not remain significant after Bonferroni correction. These results demonstrate that the constructed model did not rely on the strength of a single functional network, but rather it incorporates information related to motor impairment from various neural networks throughout the brain.

**TABLE 3:** Predictions from lesioned models constructed from drug‐naïve patients and validated on drug‐managed patients

		Drug‐naïve group		Drug‐managed group	
		*r*, *p*_permu_	*Z*, *p*	*r*, *p*	*Z*, *p*
	Whole brain	.845, .002	—	.209, .025	—
Lesioned model	−FP	.811, .001	2.149, .032	.202, .031	0.205, .837
	−DM	.845, .001	0, 1.0	.202, .030	0.535, .592
	−Mot	.809, .002	1.426, .154	.147, .117	1.110, .267
	−Vis	.819, .003	2.246, .025	.209, .024	0.961, .049
	−Lim	.836, .001	0.587, .557	.195, .036	0.639, .523
	−BG	.845, .001	0, 1.0	.192, .039	2.232, .026
	−Cer	.843, .001	0.695, .392	.203, .029	0.838, .402

*Note*: Predictability of models constructed from drug‐naïve patients were generated with consensus connections and remained significant by using the optimal threshold of model M (*p* = .009). Predictability was assessed by calculating Spearman correlation coefficients (*r*) between observed and predicted scores. The significance of the prediction was confirmed by permutation testing in drug‐naïve patients (*p*_permu_). Predictability from whole‐brain matrix is included in the first row for comparison. Results of Steiger's tests did not remain significant after Bonferroni correction.

Abbreviations: BG, basal ganglia network; Cer, cerebellum network; DMN, default mode network; FP, frontoparietal network; Lim, limbic network; Mot, motor network; Vis, visual‐related network.

#### 3.5.2 Investigating connection patterns of the constructed model

**FIGURE 4:**
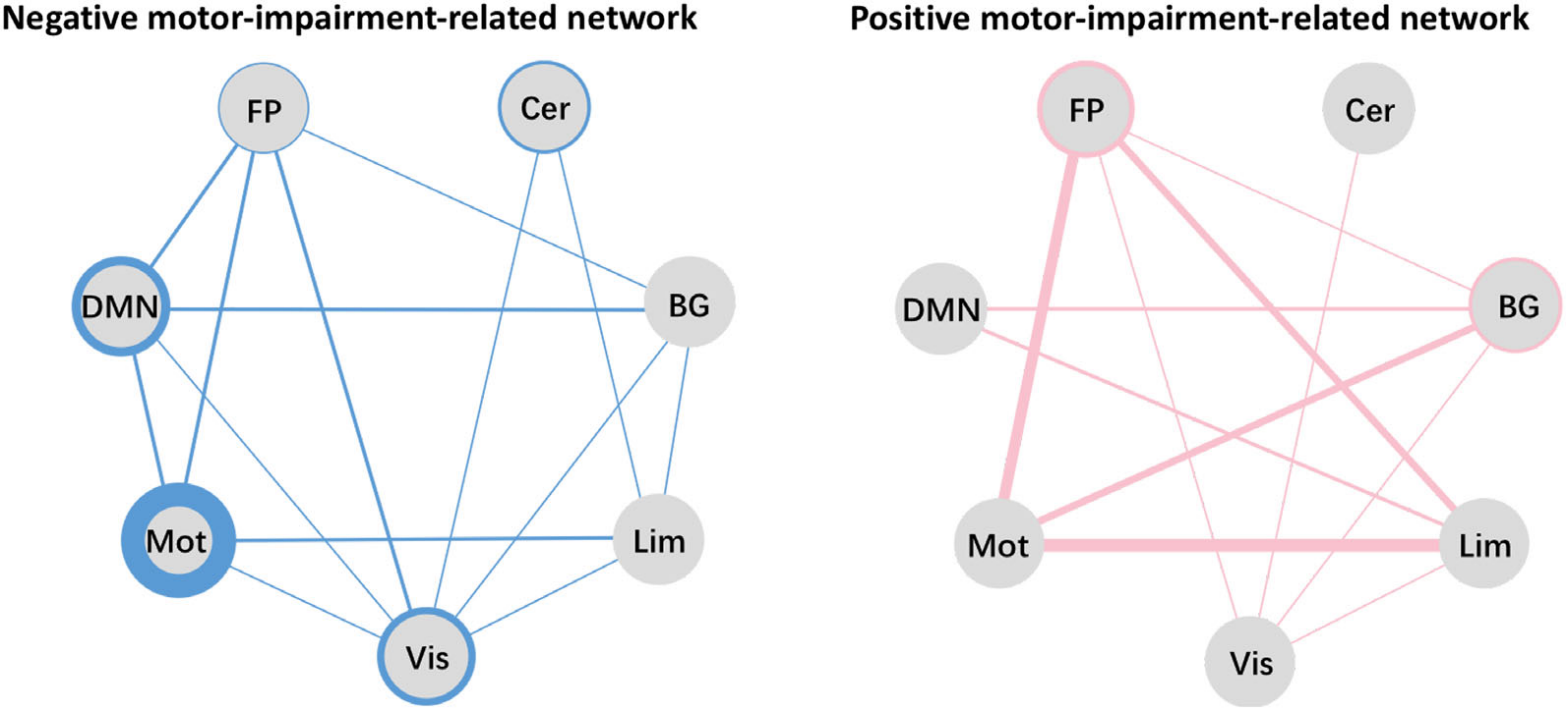
Negative (left, blue) and positive motor‐impairment‐related networks (right, pink). To control for the possible effects of network size, the proportions of the within‐ and between‐network connections were obtained by dividing the actual number of connections by the total number of all possible connections. Each solid circle represents a functional network; thicker circles and lines represent a greater proportion of connectivity. BG, basal ganglia network; Cer, cerebellum network; DMN, default mode network; FP, frontoparietal network; Lim, limbic network; Mot, motor network; Vis, visual‐related network

We then examined the connection patterns within and between the seven functional networks in the positive and negative motor‐impairment‐related networks by taking network size into consideration and obtaining the proportion of connections that each network contributes (Figure 4).

Results showed that, in the negative motor‐impairment‐related network, the motor, visual‐related, and default mode networks were the top three contributors of within‐network connections to this pattern. This reflected the more segregated pattern for this network, which contained more within‐network connections (26.35‰) than between‐network connections (20.57‰; Table S1). By contrast, in the positive motor‐impairment‐related networks, connections between motor‐frontoparietal, motor‐basal ganglia, and motor‐limbic networks were the highest contributions. This indicates a more integrated pattern for the positive motor‐impairment‐related network, involving more between‐network connections (28.62 vs. 5.02‰ for within‐network connections; Table S1).

These results demonstrate that more within‐network connections among motor, visual, and default mode networks were indicative of lower UPDRS III scores and mild PD motor impairment, whereas more between‐network connections among motor‐frontoparietal, motor‐basal ganglia, and motor‐limbic networks were associated with higher UPDRS III scores and severe PD motor impairment.

## 4 DISCUSSION

By applying a data‐driven CPM method, a resting‐state functional‐connectome‐based model was constructed to predict the severity of motor impairment in drug‐naive patients and to characterize PD motor impairment in terms of coordinated functional activity across distinct networks. Moreover, this model, constructed from data on drug‐naïve patients, could predict motor impairment in drug‐managed patients.

According to the correlation between the connection strength and the UPDRS III scores, we assigned all selected connections into one of two patterns: negative and positive motor‐impairment‐related networks. In the negative motor‐impairment‐related network, the strength of each connection was inversely associated with UPDRS III score, that is, higher UPDRS III scores (more severe motor impairment) were correlated with a lower sum of strength in this network. On the contrary, more severe motor impairment also correlated with a higher sum of strength in the positive motor‐impairment‐related network. By comparing the connection patterns in these two networks, we found that the negative motor‐impairment‐related network was predominantly constructed by within‐network connections, a reflection of the higher segregation in distinct networks. Conversely, the positive motor‐impairment‐related network was principally characterized by between‐network connections, which suggests highly integrated communication across different distinct networks (Damoiseaux, 2017; King et al., 2018). Thus, these results show that motor impairment in PD is related to two different connection patterns: mild impairment is associated with a more segregated pattern, whereas severe impairment is linked to a more integrated pattern. Our findings are consistent with those of Kim et al. (2017), who, by employing dynamic functional connectivity, found that PD patients exhibited two states. State I is characterized predominantly by functional connectivity within regions belonging to a specific network, and in State II functional connectivity is principally between regions belonging to different networks. Furthermore, the severity of motor impairment was positively correlated with loss of segregation and enhanced interactions between distinct networks (Kim et al., 2017; Nieuwhof & Helmich, 2017). Therefore, alongside previous findings, it can be suggested that the decreased functional segregation and increased functional integration are critical in modulating motor impairment in PD.

Next, we investigated the predictive contribution of each functional network in the negative motor‐impairment‐related network, which was mainly composed of connections within the motor, visual, and default mode networks. Thus, dysfunction within these three networks might accelerate motor impairment in PD. Furthermore, of these connections, those in the motor network were the most important. Similarly, a number of previous studies also suggested that intrinsic dysfunction of the motor network had a close relationship with motor deterioration in PD (Lewis & Byblow, 2002; Tessitore, Giordano, De Micco, Russo, & Tedeschi, 2014; Wu et al., 2009), even in patients at different stages of the disease (Tessitore et al., 2019). In addition to the motor network, the visual‐related and default mode networks were shown to contribute significantly to the negative motor‐impairment‐related network. Visual dysfunction is a major symptom of PD, manifesting as loss of visual acuity and color vision as well as higher‐order visual deficits (Weil et al., 2016). The interaction between the visual and motor networks is important for learning and controlling movements (Glickstein, 2000) and is known to be impaired in PD patients (Inzelberg, Schechtman, & Hocherman, 2008). Furthermore, a significant negative correlation between connection strength within the visual network and the severity of motor symptoms had been demonstrated among PD patients with freezing of gait (Tessitore et al., 2012). The default mode network is the most studied network, and has the highest number of network connections, and exhibits complex neural modulations (Mohan et al., 2016). Specifically, two studies demonstrated that the default mode network participates in motor coordination and cognitive function, both of which are closely related with motor modulation (Fox & Raichle, 2007; Greicius, Krasnow, Reiss, & Menon, 2003). Pathologically, α‐synuclein deposition, along with the disruption of dopaminergic pathway, might affect the modulation between default mode network activity and other networks, leading to motor impairment (Christopher et al., 2015). Taken together, this negative motor‐impairment‐related network, composed mostly of within‐network connections of the motor, visual, and default mode networks, provides insightful evidence that motor impairment in PD is caused by dysfunction of these networks, which could not be simply ascribed to the motor network.

Conversely, the positive motor‐impairment‐related network was mainly composed of connections between the motor‐frontoparietal, motor‐basal ganglia, and motor‐limbic networks. Therefore, based on the disruption of the motor network in PD as mentioned above, the increased connection between the motor network and other networks, for example, the frontoparietal, basal ganglia, and limbic networks might indicate their enhanced activation to protect the motor network from pathophysiological dysfunction, leading to an amplified motor modulation. Other than the enhanced connection between the motor and basal ganglia networks that have been associated with the severity of motor impairment (Kwak et al., 2010; Tessitore et al., 2019), this study has also identified a potential compensation mechanism that the brain uses to overcome motor impairment by increasing the communication between the motor‐frontoparietal and motor‐limbic networks. Increased functional activity within the frontoparietal and limbic networks has been reported to be related to PD motor symptoms such as hypokinesia/akinesia (Martin et al., 2019), freezing of gait (Bartels & Leenders, 2008; Shine et al., 2013), and “masked face” syndrome (Rizzo et al., 2018). The frontoparietal network is considered to be one of the top‐down control networks involved in initiating and adjusting control (Dosenbach, Fair, Cohen, Schlaggar, & Petersen, 2008). Meanwhile, the limbic‐motor connections were also found to be involved in the emotional adjustment of complex functions such as spatial perception and movement computation (Rizzo et al., 2018). The enhanced functional communication of motor‐frontoparietal networks and motor‐limbic networks detected in the positive motor‐impairment‐related network supports the observation that PD patients may rely on more attentional and emotional resources to overcome their motor dysfunction due to a loss of automaticity.

Because PD is clinically heterogeneous and drug uptake can influence the organization of the brain, the constructed CPM model should be validated in drug‐managed patients. Accordingly, the significant prediction was validated after translating the CPM model constructed with drug‐naïve patients to drug‐managed patients. Although all drug‐managed patients were required to be free of drugs for 12 hr, the long‐duration response to levodopa can persist for several days after drug cessation; this has been demonstrated to affect cortical function involving motor control (Cilia et al., 2020; Donzuso et al., 2021). The successful validation in drug‐managed patients demonstrated that this selected functional connectome representing the intrinsic organization in PD is preserved after chronic levodopa treatment. Although within‐subject comparisons between pretreatment and posttreatment status would be a stronger test of this hypothesis, the current results provide compelling evidence that these generalizable brain–behavior associations were independent of the effect of dopamine.

In summary, our findings suggest that these disease‐intrinsic connectome characteristics identified in drug‐naïve patients at the individual level have the potential to be a stable biomarker for the severity of motor impairment in PD. The reduced predictive power in the drug‐managed group compared with drug‐naïve patients also demonstrated that chronic levodopa treatment might influence the connection patterns detected, thus affecting prediction performance. Further study is needed to determine the alterations in connectivity caused by chronic levodopa treatment.

This study has several limitations that should be acknowledged. First, most patients enrolled in this study were at a relatively early stage of disease, and therefore the model is still to be validated in patients with advanced PD. Second, the current results were obtained from a cross‐sectional study; a longitudinal study is needed to validate the repeatability of the connectome as the disease progresses. Third, as a previous study suggested, the application of multimodal brain data might improve the predictive performance (Helmich, Vaillancourt, & Brooks, 2018).

## 5 CONCLUSION

This study identified a whole‐brain connectome‐based model that could predict the severity of motor impairment among drug‐naïve patients; this was further applied in an independent drug‐managed group. The connectivity patterns generated with our model suggest that functional segregation of motor, default mode, and visual‐related networks plays an important role in motor impairment in PD and that the higher coupling of motor‐frontoparietal, motor‐basal ganglia, and motor‐limbic networks might represent a compensatory mechanism to overcome motor dysfunction. This generalizable brain–behavior association can be detected in relation to pretreatment and posttreatments, indicating a relatively stable estimation of motor impairment severity in PD.

## CONFLICT OF INTEREST

The authors declare no conflict of interest.

## ETHICS STATEMENT

This study was approved by the Medical Ethic Committee of Second Affiliated Hospital of Zhejiang University School of Medicine.

## CONSENT TO PARTICIPATE

All participants signed the informed consent forms according to the Declaration of Helsinki.

## Supporting information


**Appendix** **S1**. Supporting Information

## Data Availability

The materials used and/or analyzed during the current study are available from the corresponding author on reasonable request.
