# Supplementary material for: Identifying a whole‐brain connectome‐based model in drug‐naïve Parkinson's disease for predicting motor impairment
Source: Hum Brain Mapp. 2021 Dec 31;43(6):1984–96. doi: 10.1002/hbm.25768 (PMC8933250; doi:10.1002/hbm.25768)
Supplement: Supplementary file 1 — Appendix S1. Supporting Information [file HBM-43-1984-s001.docx]

**
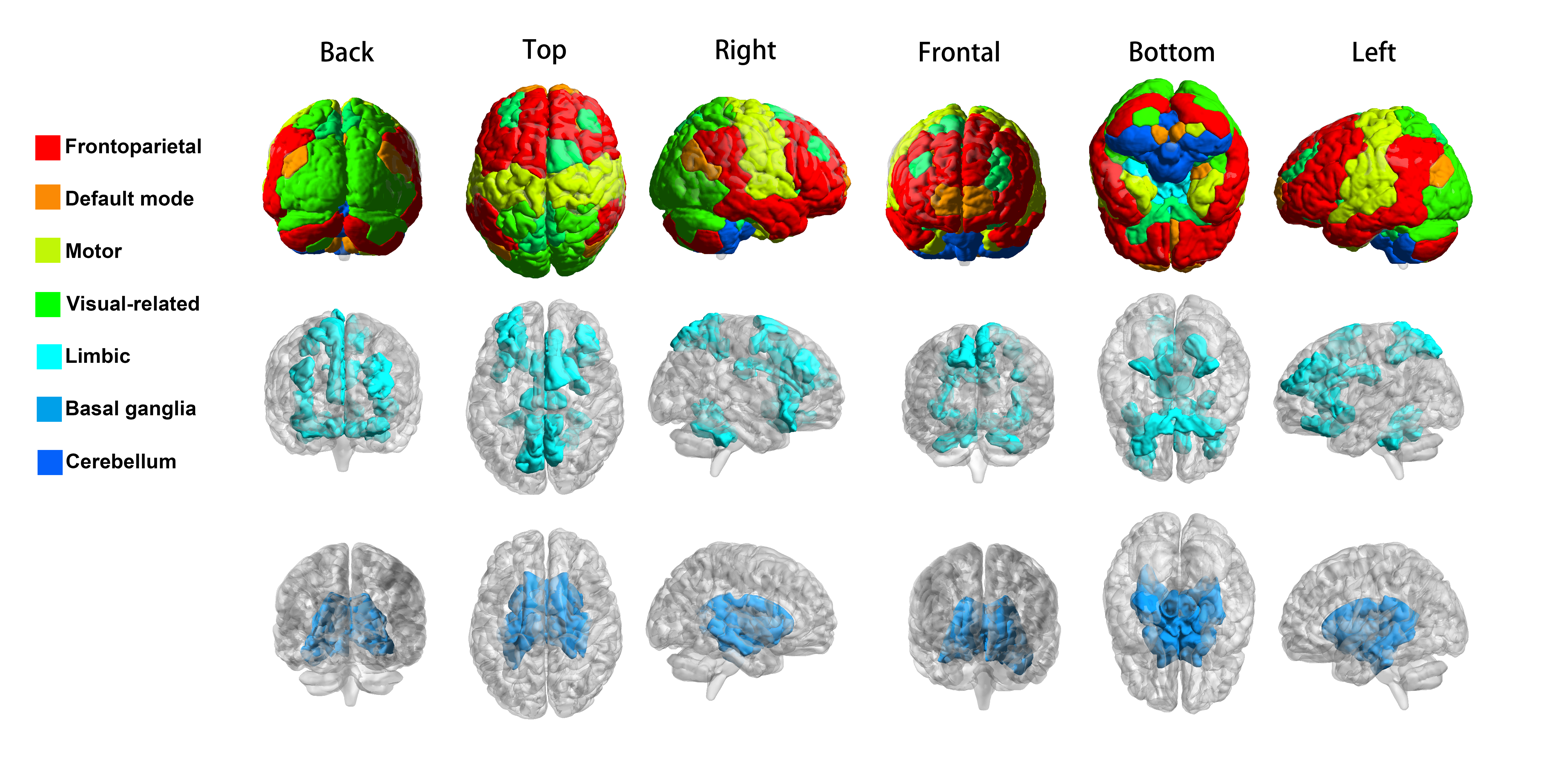
Supplemental Information**

**Figure S1. Seven** **canonical networks.** Figures were created using **BrainNet Viewer (Xia et al., 2013).**

**Reference**

Xia, M., Wang, J., He, Y., 2013. BrainNet Viewer: a network visualization tool for human brain connectomics. PLoS One 8, e68910.


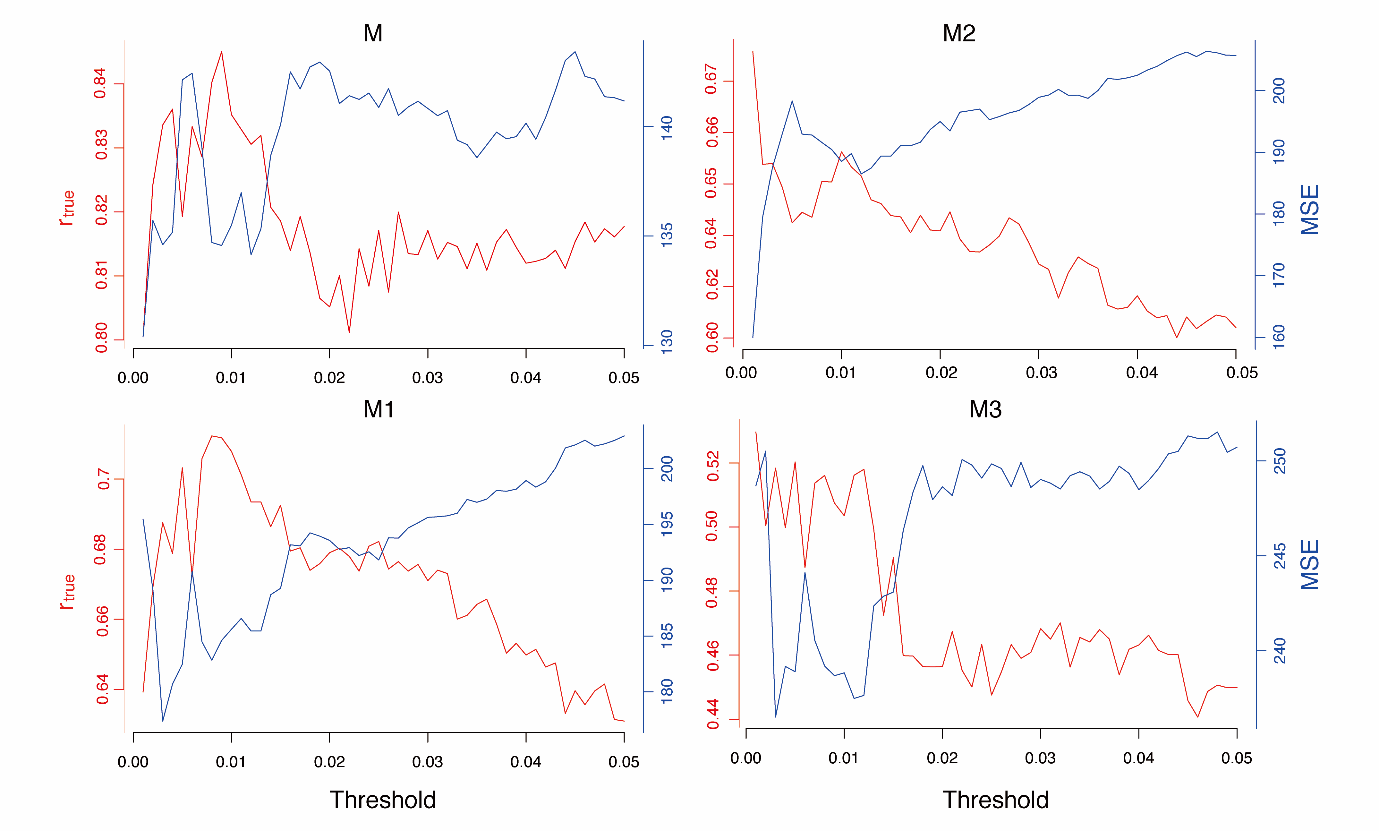


**Figure S2**. **Optimal threshold selection for different connectome-based predictive models.** The red and blue lines presented the r_true_ and MSE values across a range of p-values, respectively. The optimal thresholds of M, M1, M2, and M3 were 0.009,0.008,0.001 and 0.001, respectively.

Abbreviations: r_true_: the true predictive correlation coeﬃcient between predicted and observed scores; MSE: mean squared error.


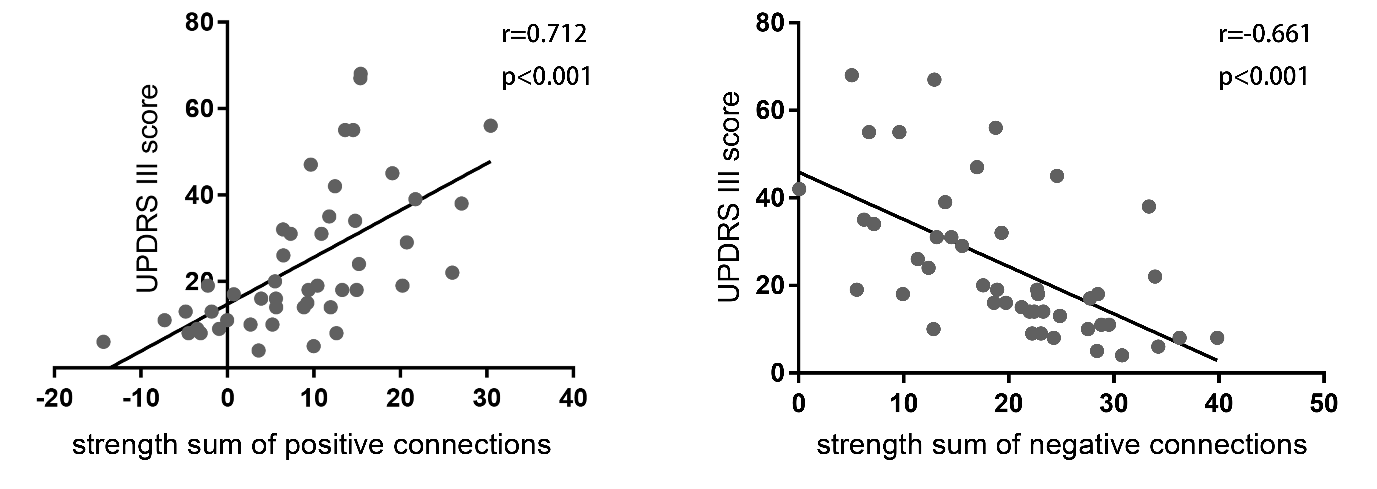


**Figure S3. Correlation between strength sum of positive/negative connections and UPDRS III scores in drug -naïve group.**

Abbreviations: UPDRS III: The Uniﬁed Parkinson’s Disease Rating Scale part III.


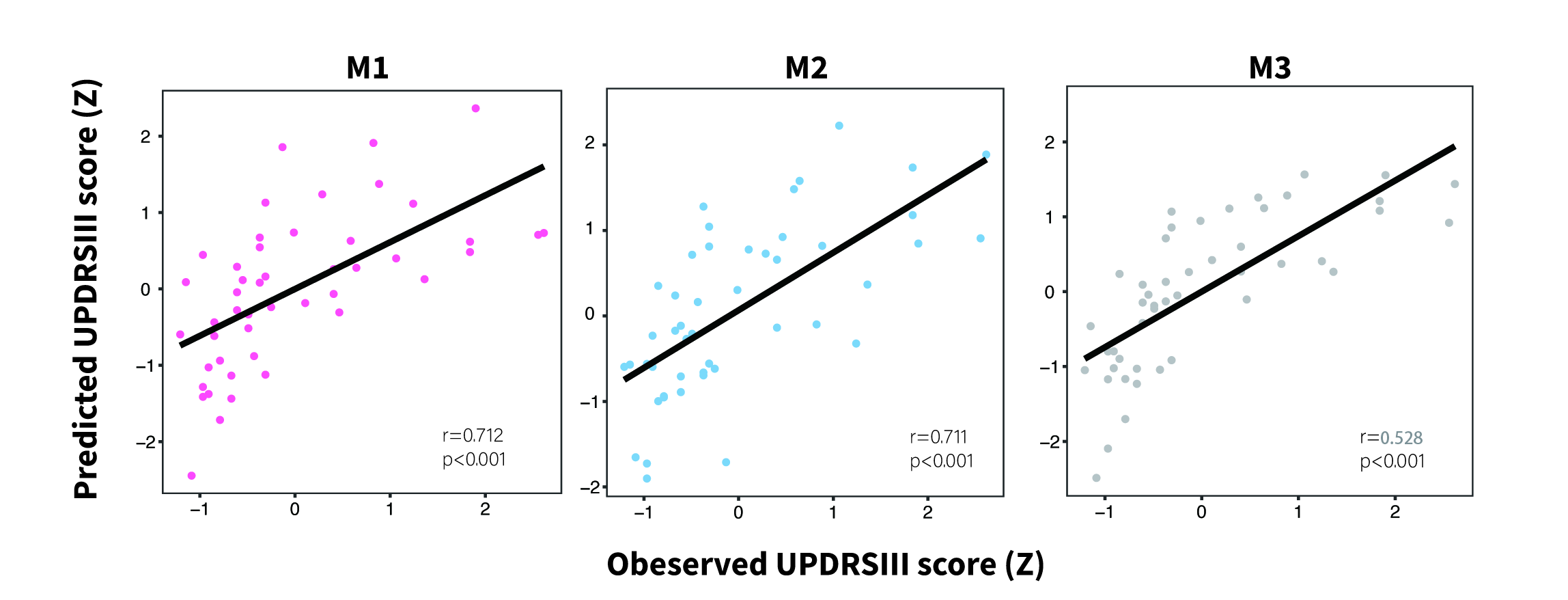


**Figure S4. Prediction of M1, M2, and M3 among drug-naïve patients.** Predicted scores generated from these three models were significantly associated with observed UPDRS III. Both predicted and observed scores were standardized for visualization.

Abbreviations: UPDRS III: The Uniﬁed Parkinson’s Disease Rating Scale part III.

**
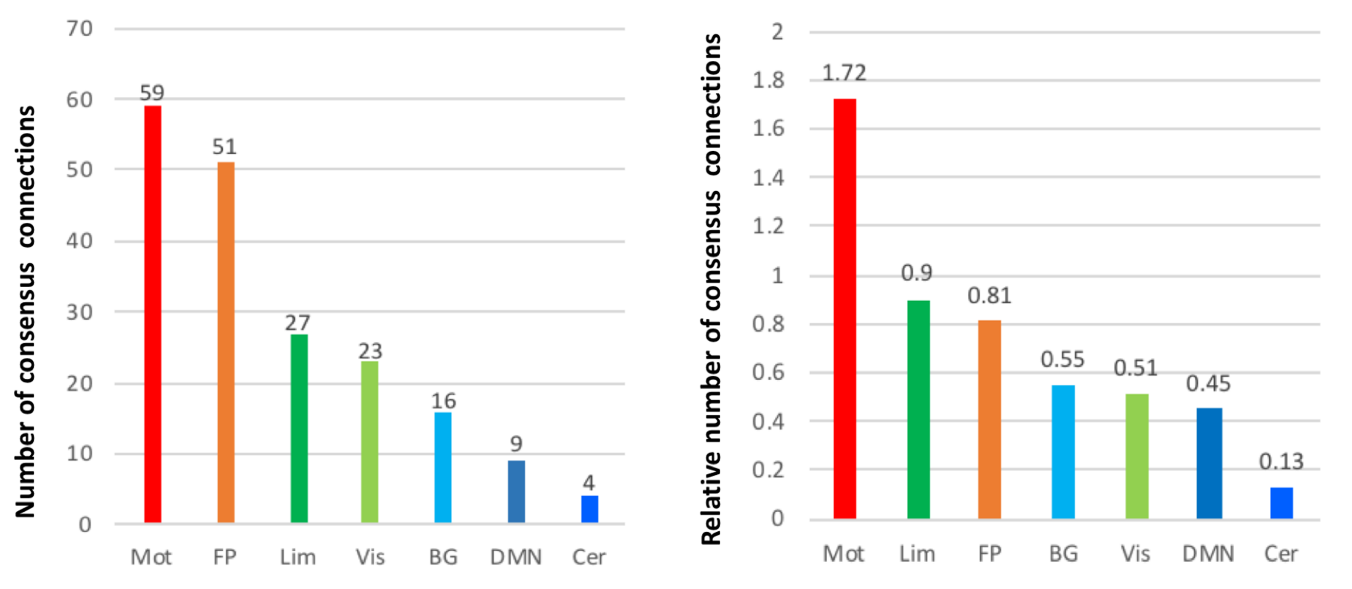
**

**Figure S5. Contribution of each functional network to motor impairment prediction.**  In order to control the effect of different network sizes (number of nodes in that network), the relative number of functional networks was also calculated (the number of consensus connections in each network/network size of each network).

Abbreviations: FP: frontoparietal network; DMN: Default mode network; Mot: motor network; Vis: visual-related network; Lim: limbic network; BG: basal ganglia network; Cer: cerebellum network;

**Table S1. Within- and between- networks connections of two connection patterns**

| Negative motor-impairment-related network | | | Positive motor-impairment-related network | | |
| --- | --- | --- | --- | --- | --- |
| Within-network connections | | | Within-network connections | | |
| Network | Number | Proportion (‰) | Network | Number | Proportion (‰) |
| FP | 2 | 1.02 | FP | 5 | 2.56 |
| **DM** | 1 | 5.26 | DM | 0 | 0 |
| **Mot** | 17 | 13.88 | Mot | 0 | 0 |
| **Vis** | 4 | 4.04 | Vis | 0 | 0 |
| Lim | 0 | 0 | Lim | 0 | 0 |
| BG | 0 | 0 | BG | 1 | 2.46 |
| Cer | 1 | 2.15 | Cer | 0 | 0 |
| Sum | 25 | 26.35 | Sum | 6 | 5.02 |
| Between-networks connections | | | Between-networks connections | | |
| Network | Number | Proportion (‰) | Network | Number | Proportion (‰) |
| FP-DM | 2 | 1.58 | FP-DM | 0 | 0 |
| FP-Mot | 6 | 1.90 | **FP-Mot** | **19** | **6.03** |
| FP-Vis | 5 | 1.76 | FP-Vis | 1 | 0.35 |
| FP-Lim | 0 | 0 | FP-Lim | 7 | 3.7 |
| FP-BG | 2 | 1.09 | FP-BG | 2 | 1.09 |
| FP-Cer | 0 | 0 | FP-Cer | 0 | 0 |
| DM-Mot | 2 | 2.00 | DM-Mot | 0 | 0 |
| DM-Vis | 1 | 1.11 | DM-Vis | 0 | 0 |
| DM-Lim | 0 | 0 | DM-Lim | 1 | 1.67 |
| DM-BG | 1 | 1.72 | DM-BG | 1 | 1.72 |
| DM-Cer | 0 | 0 | DM-Cer | 0 | 0 |
| Mot-Vis | 5 | 2.22 | Mot-Vis | 0 | 0 |
| Mot-Lim | 3 | 2 | **Mot-Lim** | 11 | 7.33 |
| Mot-BG | 0 | 0 | **Mot-BG** | 6 | 4.14 |
| Mot-Cer | 0 | 0 | Mot-Cer | 0 | 0 |
| Vis-Lim | 2 | 1.48 | Vis-Lim | 1 | 0.74 |
| Vis-BG | 1 | 0.77 | Vis-BG | 1 | 0.77 |
| Vis-Cer | 1 | 0.71 | Vis-Cer | 1 | 0.72 |
| Lim-BG | 1 | 1.15 | Lim-BG | 0 | 0 |
| Lim-Cer | 1 | 1.08 | Lim-Cer | 0 | 0 |
| Cer-BG | 0 | 0 | Cer-BG | 0 | 0 |
| Sum | 33 | 20.57 | Sum | 51 | 28.26 |

Note: The top three within- or between-connections of negative and positive motor-impairment-related networks were bolded. The proportion of within and between networks connections was calculated by dividing the actual number of connections by the total number of all possible connections.

Abbreviations: FP: frontoparietal network; DMN: Default mode network; Mot: motor network; Vis: visual-related network; Lim: limbic network; BG: basal ganglia network; Cer: cerebellum network;

**Construction processes of M1, M2 and M3**

**1.M1: Model constructed with consensus positive connections**

Consensus positive connections were defined as positive connections that overlapped in each iteration of the leave-one-out cross-validation (LOOCV). M1 was constructed with consensus positive connections retained significant by using the optimal threshold. The optimal threshold here was defined as the one leading to the highest r_true_ of models only enrolling consensus positive connections for model construction. The sum of strength of these connections was seen as an independent variable and fitted into the general linear model relating to the observed UPDRS III score. After that, the predicted score of each patient could be calculated by applying the constructed model with the following formula:

$$predicted score=c_{1}\times x_{1}+d_{1}$$

$x_{1}$=sum of strength of consensus positive connections

**2. M2: Model constructed with consensus negative connections**

Consensus negative connections were defined as negative connections that overlapped in each iteration of LOOCV. M2 was constructed with consensus negative connections retained significant by using the optimal threshold. The optimal threshold here was defined as the one leading to the highest r_true_ of models only enrolling consensus negative connections for model construction. The sum of strength of these connections was seen as an independent variable and fitted into the general linear model relating to the observed UPDRS III score. After that, the predicted score of each patient could be calculated by applying the constructed model with the following formula:

$$predicted score=c_{2}\times x_{2}+d_{2}$$

$x_{2}$=sum of strength of consensus negative connections

**3.M3: Model generated predictive score from each iteration of LOOCV**

In model M3, the predicted score of each subject (N=1,the leave-out one) was generated by taking the data from all other subjects as training data set (N=46) in an iterative manner. In each iteration, the model was constructed by fitting the strength sum of both positive and negative connections into general line regression. Each positive or negative connection retained significance by using the optimal threshold. Finally, the model was used to 1predict the leave-out one based on his or her own network strengths. There was a total of 47 iterations, and each of the 47 subjects was left out once.

$$predicted score=a_{3}\times x_{1}+a_{4}\times x_{2}+b_{1}$$

$x_{1}$= sum of strength of negative connections of leave-out one,$x_{2}$= sum of strength of negative connections of leave-out one;$a_{3}$,$a_{4}$ and b_1_ were generated from general line regression that applied all other subjects as training data set (N=46)
